# Daily Jottings in the Gold Book of a Dentist—January 1, to March 1

**Published:** 1851-04

**Authors:** E. Townsend


### ARTICLE IV.

### *Daily Jottings in the Gold Book of a Dentist—January* 1, *to March* 1. By E. TOWNSEND, D. D. S.—No. 1.

To-day I have had three cases worth a passing notice, and from two of them some reflections may be suggested which would perhaps modify very much, if not improve the practice of many of those who undertake the treatment of teeth without sufficient regard to their pathological condition, and their anatomical structure. I will briefly state these cases, and explain the mode of treatment which I have commenced, and in which, if I am successful to the end, I hope to produce a state of such comparative health to the organs as to make them useful for many years. The first is a front incisor, which the patient, a lady, applied to a dentist, and one in high repute too, to have filled; finding it somewhat tender, and probably supposing the membrane exposed, he used the actual cautery, to such an extent as to penetrate to the membrane, and then immediately filled the tooth. The lady complained of great soreness of the tooth and surrounding teeth, and in a few days a change of color was very apparent, still, hoping it was a temporary difficulty which would soon subside, delayed asking advice till I saw her, when the pain had become almost unendurable. I immediately removed the filling, which indeed was not difficult, and a gush of pus from the orifice showed but too plainly the irritation which had been caused by this injudicious operation. Relief from pain was the immediate consequence of this liberation of pus, I shall see her every day, washing out the fang at each visit and directing it to be kept free from any extraneous matter.

Case No. 2, was one in which the operator had filled a front incisor over the exposed pulp, which, though the orifice was so small as to be scarcely visible, and had to be enlarged for the free entrance of a small probe, was still sufficient to destroy the vital connection between the internal circulation and the bone, and the death of the tooth and inflammation of the periosteum was the inevitable result. The dentist, in this case, was consulted repeatedly, and attempted to alleviate, by lancing freely, and even by actual cautery of the gum—without, perhaps, ever thinking that he had dammed up the whole cause of trouble within the fang, and as long as that reservoir remained, the external opening was as necessary, and as impossible to heal, as it would be to heal healthily the skin of any part of the body over an ichorous and suppurating surface.

I removed the filling, which was compact and firm, and found the fang filled with bloody serum; injected with warm water, and directed it to be kept free and clean; shall fill the fang to the apex, when it becomes entirely healthy.

Case No. 3.—A large inferior molaris, right side, had been filled, several years since, with amalgam, as the dentist assured the lady *he* could not fill it with any thing else. Some time after, inflammation and pain set in, and another dentist was consulted, who said it was the poisonous nature of the vile compound with which it was filled, which had caused all the trouble, and proposed, when the inflammation should subside, to extract the tooth, as the only means of CURE.

When the attack had passed off, the lady again called on him, but was unwilling to submit to the loss of the offending member, and nothing was done, as he said the tooth could not be refilled if the poisonous material *were* removed, which it would be very difficult to do. A few months after, the lingual portion of the tooth broke away, and the amalgam fell out; and in this state she called for my advice, expecting to have it extracted. After close scrutiny, I resolved to attempt to save it, and upon passing a probe down both the fangs, found there was still some vitality in them; although there had been inflammation, there had been no abscess, and therefore there was probably still sufficient recuperative power in the surrounding parts and anastomosing radial vessels, to secure a favorable result. I introduced a pledget of cotton, saturated with chloride of zinc, and to-morrow shall hope to find that the portions of sensitive membrane remaining will come away with a clean slough. This tooth is somewhat discolored through the entire dentine, by the mercury and silver, and the patient supposed, for so it was represented to her, all the trouble she had, and all the inflammation, was in consequence of the deleterious nature of the compound with which the tooth was filled—when the very person who so inveighed against it, knew perfectly well, that any other stopping, equally impervious, would have produced the same effect—that even a pledget of cotton, if pressed firmly into the cavity of a tooth predisposed to periodentitis, will produce pain of a violent character. Why not make their objections to *any* inferior stoppings for teeth, on the true ground. When will the men in our profession, to whom we *should* look for instruction and example, be true—true to themselves, to their profession, to all their brethren *in* the profession, whether high or low, if he have one talent to answer for or five? When will they learn, that to condemn a practice which they would reprobate, they must take care they have truth and fairness on their side, else they may put a whip into the hand of their brother, to scourge their own back. In this very warfare about amalgam, we have had it said, there was great tumefaction of the gums, an ichorous discharge from them, and confined to the teeth in which the filling had been placed, leading directly to the inference, that the mercury had produced all this effect. Now, if ptyalism were induced by the mercury of the compound, its effect would be infused through the whole system, and its action on the gums would be sympathetic and general, not confined to one or two or three teeth, but would affect the whole mucous membrane. Those who have made use of such arguments as these, seem to have forgotten, that if they attempted to prove too much, and the advocates for its use conclusively show these effects produced by other means—then, if their premises were false in *this*, why not in the whole matter; and so by gratifying their love of vituperation, for in some cases it seems to have been more from this feeling than an honest interest in the general good, they have lost their labor and missed their aim. There *are* true, and honest, and conscientious, and moral grounds enough upon which to condemn the use of all *inferior* materials for filling teeth. Take amalgam of mercury and silver as an instance of this; it is always to some extent, often to a very considerable one, oxydizable in the acids of the mouth; it is not impermeable; caries often goes on under it; it is black, and almost always gives a black or blue tinge to the teeth in which it is placed. Here are reasons enough to condemn it, and we do not need to look for any more; yet there is a higher and better ground, upon which, perhaps, most will agree. Do not all who resort, even occasionally, to inferior materials for filling teeth, lose their artistic feeling in the matter? feel less and less, as they indulge in it, that they are honorably laboring for a high end, do they not in time come to consider the *fee* to be *the* thing of importance to be secured, and the salvation of the teeth, and the permanence and beauty of their work, becomes a secondary object. This is indeed bowing down to the golden calf; it is laying on its altar our honesty, our morality, and all our high hope of great achievement. There is, in the labor of filling teeth, particularly in the more difficult departments of the art, great room and scope for moral culture, and I contend, that just in proportion as the best material known is used, just in such ratio will be the perfection of the whole operation. I do not believe the same time and care *can* be taken to prepare a cavity for a filling, where we know, in consequence of the imperfectability of the material to be employed, the operation must needs be of short duration of usefulness.

There is, in the mind of man, a great distaste to doing any thing that is not to serve some end, and may we not say, a natural honesty, that prevents him from doing that with his whole heart, which does not benefit some one. I once heard an anecdote which very happily illustrates this: a poor man called upon a gentleman for some assistance, saying he would be very glad to work, if he could only procure it; the gentleman benevolently desiring to assist him, though he had really nothing that he needed he should do, told him he might remove a pile of stones he pointed out to him, from one side of the road where they were, to the opposite side. The man went to work, and diligently, and in the course of the day removed them, and piled them nicely on the spot indicated. When he had finished and received the money for his labor, he asked his employer if he had any thing more he wished him to do, he would be very thankful for any small job his honor would give him. When told he had nothing else, except he chose to carry the stones all back again, he demurred, and said, "Inda'ad, yer honor, there is no use in *that*, at all, at all, and it would only be putting the money into me own pocket, without a hap'orth good to any body else—so, though I want work, I can't do that."

Let each man who is tempted to use an inferior stopping for a tooth, either from the ease with which it may be introduced into a difficult cavity, its cheapness, or any other self-indulgent cause, think, for one moment, whether any great good is to be gained except the fee which he dishonestly receives. If this is all, he is prostituting his calling to base uses, and can never, by any chance, be anything better than a mountebank.

But I have digressed widely from my cases, which shall be reported and jotted down as they progress.

Case No. 1.—Left open for three days, pain and soreness gradually subsiding, though the periosteal membrane was still tender to impressions of cold air; in ten days the abscess entirely disappeared, and the tooth almost regained its color—the last five days of which it was kept filled, not tightly, with cotton, which was saturated with chloride of zinc. In three weeks from the first opening, it was refilled to the end of the fang, and has since been perfectly comfortable.

Case No. 2 has also been apparently successful. After a complete and thorough cleansing of the fang, and repeated washings with warm water, it was left to free itself of its impurity, and at the end of five days, all soreness having left it, it was kept loosely stuffed with cotton, saturated with chloride of zinc, and in fifteen or sixteen days filled to the end of the fang. Upon examining the three remaining incisors, the same treatment was very plainly indicated, and they have all been treated in the same way, and with the like apparent success. The patient herself expresses the greatest satisfaction, and gives the strongest evidence that can be given of feeling of health—"that they don't feel at all," or in other words, she is not conscious of her teeth. If they are in like condition two years hence, the treatment will not have been in vain.

Case No. 3 is finished to my own satisfaction, and very much to the gratification of the lady; but time, that tests all our operations, must show whether it was well or ill advised to attempt the rescue of a molar from the forceps, which, in the opinion of one of the best operators of New York City, was fit for nothing better. On the next day after inserting my pledget of cotton and chloride of zinc, I took away, as I had hoped, the remainder of the membranes from the fangs, and in three days more, finding no inflammation supervened, I proceeded to the filling of the tooth, which, as I knew it would occupy several, perhaps five or six hours, I undertook on New Year's day—a day so generally observed as a holiday, that interruptions were not likely to be so frequent as usual. After filling both the fangs to the floor of the main cavity, and burnishing the fillings, I proceeded to build up the crown to the tooth; the inner or lingual side of the crown was broken down to the margin of the gum almost, and also round the posterior part. In filling this cavity, I made a number of pellets, of an eighth, and some of a quarter of a sheet of gold, No. 4, and packing firmly into the bottom of the cavity; after securing nearly two sheets in this way, built a wall of gold along the lingual border, and making this wall very firm, to a height rather above the outer edges of the tooth, packed into the center and along the anterior border, with gold folded in half sheets, making all the pressure directly towards the center and bottom of the cavity; after putting away between seven and eight sheets in this way, began to consolidate the surface, and to press in all the ends of the folds that had been introduced, and then burnished the whole surface carefully before allowing any saliva to moisten it. This is, perhaps, one of the most important and least heeded points in perfectness of the operation of filling teeth, for I contend that no gold can be made perfectly solid, if a particle of moisture is between the laminæ, and that, if kept entirely dry, it may be packed on, one fold on top of another, and so made to take *any* shape, or attain *any* height above the main cavity that may be desirable. I know this has been doubted by some, who contend that the folds of gold must be pressed laterally against the walls of the opening, and then a wedge-shaped instrument inserted to make room for more, and so on till the cavity is full; but is it full, if it is so soft, as it must be at the bottom, for with little or no direct and forcible pressure in the direction of the floor of the cavity, until after all or nearly all the gold is put in, it will not then yield except just at the surface, and there it may be made hard; but it is hollow and soft within, and the ordinary wear of mastication soon proves it to be so, for it sinks below the surface, and thus becomes a receptacle for food, and probably caries commences at the edges, and so the perfectability of the work is lost. So important do I consider this absence of moisture to be, that in cold weather I warm all the gold, after it is cut and folded, on a plate which is over a small spirit lamp, and also warm all the instruments used in packing the foil, thereby preventing the condensation of the atmosphere of the mouth, which, when the thermometer is at 15° or 20° in the open air, is quite sufficient to prevent gold from adhering and making a compact solid filling.

But to return to New Year's day. After the surface had been thoroughly burnished, it was thoroughly filed with smooth bent files, and then stoned with Scotch stone; after which pumice and soft wood were used, and then the whole was reburnished with a polished steel burnisher and soap. In one week the patient said the tooth, which, prior to the operation had been worse than useless, as it prevented her using that side of her mouth, felt perfectly well; she was using it freely, and soon expected to forget anything had ever ailed it.

Case No. 4.—A young lady, sixteen years of age, has been, since twelve, under the care of a so-called *surgeon* dentist, and truly he has thought to prove his title to the name, by extracting nearly all the poor child's teeth, saying they could not be filled, and nothing else was practicable but the use of the forceps, forgetting the maxim a dentist should ever remember, "to extract is not to cure, but to destroy." In this case, the central and lateral incisors on the right side, having been somewhat, though slightly, tender, in excavating preparatory to filling, arsenious acid was introduced, which allayed the pain truly, but, unfortunately, did a great deal more, for the absorbent vessels took it up and destroyed the membrane and produced discoloration of the tooth and abscess. Now this partial tenderness of the carious portion of the bone, can generally be entirely removed by dexterously cutting it away with a sharp excavator, or, if the patient is unwilling to bear the momentary pain of this, an application, for a half hour, of chloride of zinc, will almost always effect the object; if all the portions of softened bone be then carefully removed, the tooth can be filled with as much force as is necessary to make the gold perfectly solid. The question is often asked, why is the bone so tender and exquisitely painful as it often is, at the commencement of excavating, and yet sometimes entirely painless when the diseased portions of it are removed. Why should it not be attributed to inflammation. The teeth, though not highly organized, are supplied with a nerve in each fang, an artery and a vein; these give the internal vitality to the tooth, and pass that vitality through its whole bony structure, till they meet the enamel. If, then, the branches of the nerve pass through the whole bone of the tooth, to the investing surface, called enamel, it is easy to understand how the minute ends of these nerves may be exquisitely sensitive when there is caries and inflammation. It is in the experience of every one who has practiced dental surgery, that when he has cut into the pulp cavity, and severed the connection between the bony surface immediately over it and the nerve and blood-vessels, the pain which was felt before in all parts of the tooth, immediately ceases, no portion of the cavity then being tender to the excavator, but the membrane or nerve. Does not this seem to prove, conclusively, the nerve to send minute microscopic branches through the whole tooth, which are the medium of sensation as long as the connection between them and the bone is not ruptured. This has been my own theory for many years, and I have acted on this theory in my practice.

The central incisor of which I just spoke, was the immediate cause of the application for aid. Finding it filled on the approximal surface, between the central and lateral, I removed the filling, and was obliged to cut through a thick septum of bone to enter the cavity. Here I found what I expected, a total decomposition of the vessels, and an exceedingly offensive discharge; washed with warm water, and left it open, and then treated the lateral tooth in the same way, finding it in the same condition. Further examination of the mouth showed the bicuspid on the right side carious, and the inner or lingual cusp broken away; the molar on the same side also had a large cavity in its grinding surface. These were both under the ban of the *surgeon*, and yet neither of them had penetrated to the nerve cavity. They have both been filled without any other difficulty than could easily be overcome by care and perseverance. The bicuspid and remaining molar on the other side were in a much worse condition, being both decayed to the membrane; the bicuspid was broken away on the posterior portion, and after the nerves were removed, it was filled with gold, and the crown built up into its original shape. The molar, though not broken away as the bicuspid, was more difficult, as the orifice through which all the nerves were taken out, was very small; these were, however, after some difficulty, removed, and then filled, and the orifice in the crown also. The incisor teeth have been perfectly comfortable, and are much whiter than prior to the operation.

Case No. 5.—A treatment for irregularity of the superior denture. Patient twelve years of age; all the superior incisors closing within the inferior, and cuspidati somewhat prominent. This was easily remedied by a stiff hoop of gold plate, so bent as to press on the surfaces of the cuspidati, leaving space between the incisors and the hoop, of just as much as it was desired to bring them forward. The upper edge of the hoop was then filed so as to leave opposite to each of the teeth a piece of the plate to fasten a band of india rubber, which, from its elasticity and constant traction, in a week brought the teeth into the desired position, closing entirely anterior to the inferior maxillary. The india rubber loops were then removed, and ligatures of strong thread substituted, which will be changed every three days until the teeth have become perfectly firm.

To-day, have had the pleasure of seeing some fillings, in the mouth of a lady, by one of our best operators. They were well placed, and most of them firm and solid, and would have been faultless, if, (oh, how often that little word comes in to spoil our brightest dreams of perfection,) if the bicuspid teeth had been properly shaped at the edge, between the cusps; but here was what I conceive to be a great error—the separation between the teeth had been made with a knife-edge file, and the portion of enamel between the filling and the end was so thin that it has broken out, and thus exposed the ragged edge of the filling. There are no teeth that require more skill and care in the formation of the space between them, and also of the cavity to receive the filling, than the bicuspids.

Three things are absolutely necessary to success in these fillings: first, the space should, if possible, be so filed as that if it closes it shall not meet a corresponding flat surface on the adjacent tooth, i. e., shall be filed wider on the lingual than the buccal edge; second, the lower part of the cavity, or that between the cusps, shall be cut away, either with a sharp-edged cutter or a suitable file, until the border of the cavity is firm and strong; and thirdly, the upper edge, which is often under the free margin of the gum, should be very square and firm, not cut under in a deep groove, as it often is—for if so, independently of the difficulty, almost the impossibility of making that part of the filling compact—then their edges will break away, and then caries progresses, and that part of the cavity being nearer than any other to the internal membrane, *it* is soon reached, and if not then timely arrested, the entire loss of the tooth is inevitable. Many persons who fill ordinary cavities very well, fail in bicuspids; and perhaps some of them from not making as free use of the file as is necessary; in these teeth, especially, a very free use of that valuable instrument is absolutely necessary.

One of the difficulties attending this operation, is that of excluding all moisture; we often find, even after effectually preventing trouble from the duct of Steno, that we have an oozing from the gums themselves, and very frequently from the firm margin of the gum between the teeth. In some cases, this difficulty may be avoided, by using a piece of wood cut in the shape of a dove-tail, with the broadest surface to the gum; if this is forced between the teeth tightly, all danger from that quarter is prevented. In other cases, from the gum hanging down at the edges and rising in the center, like a festoon, this may be inadmissible; here a piece of soft cork may answer well, or a piece of tissue paper twisted into a rope.

The dentist who would be able to conquer all difficulties as they present, must be very fertile in expedients; every law of mechanics should be at his control; all the arcana of the sciences should be thrown open to him, and they should be his ministers, if he would be successful.

Some dentists are in the habit of filling the fangs of the teeth, after having taken out the nerves, with tin, and then filling the crown over it with gold; and others, as would appear from a case of to-day, put in a little stopper of tin, after *soothing* the nerve, and leave the membranes in the canal, to produce sure trouble afterwards. The case I speak of, was treated by a surgeon dentist in New York, of great name—but it seemed to me to have been conducted on the most approved principles of "old grannyism." The nerve was found in the canal, an offensive and putrefying mass. This was thoroughly removed, and the fang washed out clean to the apex, then filled with gold, and the crown built up into its proper shape with the same metal, the only one fit to use in a tooth.

